# A Single Angiotensin II Hypertensive Stimulus Is Associated with Prolonged Neuronal and Immune System Activation in Wistar-Kyoto Rats

**DOI:** 10.3389/fphys.2017.00592

**Published:** 2017-08-31

**Authors:** Jasenka Zubcevic, Monica M. Santisteban, Pablo D. Perez, Rebeca Arocha, Helmut Hiller, Wendi L. Malphurs, Luis M. Colon-Perez, Ravindra K. Sharma, Annette de Kloet, Eric G. Krause, Marcelo Febo, Mohan K. Raizada

**Affiliations:** ^1^Department of Physiological Sciences, College of Veterinary Medicine, University of Florida Gainesville, FL, United States; ^2^Department of Physiology and Functional Genomics, College of Medicine, University of Florida Gainesville, FL, United States; ^3^Department of Psychiatry, College of Medicine, University of Florida Gainesville, FL, United States; ^4^Department of Pharmacodynamics, College of Medicine, University of Florida Gainesville, FL, United States

**Keywords:** MRI, hypertension, inflammation mediators, angiotensin II, blood pressure, ANS

## Abstract

Activation of autonomic neural pathways by chronic hypertensive stimuli plays a significant role in pathogenesis of hypertension. Here, we proposed that even a single acute hypertensive stimulus will activate neural and immune pathways that may be important in initiation of memory imprinting seen in chronic hypertension. We investigated the effects of acute angiotensin II (Ang II) administration on blood pressure, neural activation in cardioregulatory brain regions, and central and systemic immune responses, at 1 and 24 h post-injection. Administration of a single bolus intra-peritoneal (I.P.) injection of Ang II (36 μg/kg) resulted in a transient increase in the mean arterial pressure (MAP) (by 22 ± 4 mmHg vs saline), which returned to baseline within 1 h. However, in contrast to MAP, neuronal activity, as measured by manganese-enhanced magnetic resonance (MEMRI), remained elevated in several cardioregulatory brain regions over 24 h. The increase was predominant in autonomic regions, such as the subfornical organ (SFO; ~20%), paraventricular nucleus of the hypothalamus (PVN; ~20%) and rostral ventrolateral medulla (RVLM; ~900%), among others. Similarly, systemic and central immune responses, as evidenced by circulating levels of CD4^+^/IL17^+^ T cells, and increased IL17 levels and activation of microglia in the PVN, respectively, remained elevated at 24 h following Ang II challenge. Elevated Fos expression in the PVN was also present at 24 h (by 73 ± 11%) following Ang II compared to control saline injections, confirming persistent activation of PVN. Thus, even a single Ang II hypertensive stimulus will initiate changes in neuronal and immune cells that play a role in the developing hypertensive phenotype.

## Introduction

Interplay between the renin-angiotensin system (RAS), autonomic nervous system (ANS) and the immune system (IS) has been implicated in hypertension (HTN), and cardiovascular disease (CVD) (Santisteban et al., 2013; Han et al., 2016; Hurr and Young, 2016; Wenzel et al., 2016). Activation of the IS, and particularly circulating T cells appears to be crucial in establishment of angiotensin II (Ang II) HTN (Guzik et al., 2007; Itani et al., 2016a). Elegant work by Harrison et al has shown that Ang II plays a crucial role in activation of adaptive IS, and that depletion of T cells but not the B cells ameliorates Ang II HTN (Guzik et al., 2007). The activated IS cells in circulation can have deleterious effects, as as they infiltrate peripheral and central tissues and contribute to initation of local inflammatory responses, oxidative stress, fibrosis, vascular dysfunction and stiffening, and renal damage (Young and Davisson, 2015; Wenzel et al., 2016), among others. Our recent research has shown increased release of bone marrow (BM)-derived inflammatory and progenitor cells in Ang II HTN as well as in the spontaneously hypertensive rat (SHR), hypertensive rat models characterized by increased inflammatory and RAS-depdendent responses (Santisteban et al., 2015). These pro-inflammatory BM cells have the ability to infiltrate the PVN and as such contribute to central inflammation and dysfunctional ANS in chronic Ang II-dependent HTN (Santisteban et al., 2015). Ang II can activate the IS cells directly (Kim et al., 2016) or indirectly, via activation of the ANS (Zubcevic et al., 2014). In line with the latter, recently, an association betwen increased BM sympathetic drive and elevated production and release of BM inflammatory cells (ICs) has been demonstrated in HTN and heart disease (Zubcevic et al., 2014; Ahmari et al., 2016; Sager et al., 2016; Tay et al., 2016). Thus, there seems to exist a bilateral communication between the IS and the brain, as they appear to reciprocally modulate one another. The activated IS may also be an important factor in compromising blood brain barrier (BBB) in HTN, as specific cell types and their associated cytokines, such as IL17, which are known to be increased in HTN (Madhur et al., 2010; Amador et al., 2014), have been implicated in BBB damage (Kebir et al., 2007; Huppert et al., 2010).

More recently, a novel concept of neuroplasticity in the context of HTN has been introduced. Johnson et al showed that repeted short term pro-hypertensive Ang II challenges can induce enhanced blood pressure (BP) responses to subsequent pro-hypertensive stimuli (Clayton et al., 2014; Zubcevic et al., 2014). This was attributed to the ability of Ang II to induce persistent molecular changes in the CNS, one of which is upregulation of brain RAS, which can be maintained even in the absence of the initial stimulus (Johnson et al., 2015). This adaptive response of the CNS is thought to be initially physiological, as the brain attempts to maintain homeostasis; however, in certain overwhelming conditions, for example when dealing with more than one pro-hypertensive challenge, it is possible that this plasticity becomes maladaptive, resulting in dysfunctional ANS. Similarly, sensitization of the adaptive immune responses in development of Ang II HTN has been proposed (Huppert et al., 2010). In one study, mild pro-hypertensive stimulus appeared to sensitize IL17-expressing ICs to activation by subsequent modest Ang II stimuli, which then contributed to development of HTN (Itani et al., 2016b). This suggests an additional role for Ang II in development of immunologic memory in HTN.

Considering the apparent complexity of interplay between RAS, ANS and IS, we investigated the link between Ang II-induced immune memory and neural sensitization in control of BP. Here, we show that even a single bolus injection of Ang II enhances PVN neuronal activity and activates the peripheral and central IS, which persists long after BP normalizes. This is also associated with a delayed heightened response of sympathetic drive at the beginning of the dark cycle in Ang II-exposed rodents, several hours following normalization of Ang II-elicited acute BP increase. This suggests that sensitization of ANS may occur even after a single Ang II challenge. We propose that this is linked to activation of central and peripheral IS, reflected in elevated IL17- and activated microglia responses.

## Methods

### Animals

Adult male Wistar-Kyoto (WKY) rats aged 12–14 weeks (280–320 g, from Charles River Laboratories) were housed in a temperature-controlled room (22°–23°C) with a 12:12-h light-dark cycle. All experimental procedures were approved by the University of Florida Institutional Animal Care and Use Committee, and followed all applicable NIH guidelines. A total of 80 rats was utilized in all experiments. All experiments were performed once.

### Angiotensin II administration

WKY rats were assigned to subgroups (*n* = 6) to receive either a single acute injection of angiotensin II (Ang II) (36 μg/kg I.P., Bachem), a reportedly subpressor dose when applied chronically (Yasujima et al., 1989), or normal (0.9%) saline I.P., at 11 a.m.

### Radiotelemetry blood pressure measurements

Radiotelemetry transmitters (DSI) for chronic measurements of BP in conscious animals were implanted in adult male WKY rats, as previously described (Zubcevic et al., 2014; Santisteban et al., 2015), and allowed to recover for 7 days. Immediately prior to injections, a 60-min BP recording was performed to obtain the baseline values. Following this, all rats were injected with either a single I.P. injection of Ang II (36 μg/kg) or normal saline. All injections were performed at 11 a.m. Radiotelemetry recordings started 10 min following the injections, and continued for 5 min every 20 min over 24 h. The values for both BP and heart rate (HR) were averaged for every 1 h of recording. Spectral analysis of BP waveform signal was performed in order to derive ANS variables, using the following frequency bands for rat: very low frequency at 0–0.27 Hz (VLF, indicative of humoral effects on sympathetic drive), low frequency at 0.27–0.75 Hz (LF, indicative of overall vasomotor drive), and high frequency at 0.75–3.3 Hz (HF, indicative of cardiac parasympathetic activity), as previously described (Waki et al., 2006; Zubcevic et al., 2014; Santisteban et al., 2015). The said variables were mathematically derived using the *HeyPresto!* software as previously described (Zubcevic et al., 2014). Folowing the measurements, one group of rats was euthanized at 1 h, and another group at 24 h post-Ang II injections (*n* = 6 per group). Blood and brains were collected for fluorescence-activated cell sorting (FACS) in blood, and inflammatory cytokine quantification in the punched out PVN, as detailed below.

### Quantification of systemic and central immune responses in the wky following acute Ang II injections

Following telemetry, fluorescence activated cell sorting (FACS), as previously described (Zubcevic et al., 2014; Santisteban et al., 2015), to quantify the levels of circulating T cells and macrophages (T cells: CD4^+^, CD8^+^, CD4^+^/CD25^+^, CD3^+^/CD45^+^, CD4^+^/IL17^+^, and macrophages: CD68^+^), and angiogenic progenitor cells (CD4^−^/CD5^−^/CD8^−^/CD90^+^), previously shown to be important in Ang II-dependent HTN (Jun et al., 2012; Zubcevic et al., 2014; Santisteban et al., 2015). In addition, inflammatory cytokine quantification in the PVN was assessed using Proteome Profiler Rat Cytokine Array Kit, Panel A (R&D Systems cat#ARY008), following the manufacturer's protocol. PVN tissue was excised and dissociated in 1% Triton-X 100 PBS + protease inhibitor cocktail (P8340-5 ML), followed by centrifugation at 10,000 × g for 5 min. Samples were stored at −80°C until assay. Supernatant sample protein quantification was carried out by Bradford assay (BioRad). Samples were pooled pooled within each group and ran together on a separate membrane (total = 4 membranes). Manufacturer's protocol modification for LI-COR detection was followed, using IRDye 800 CW Streptavidin (LI-COR cat#925-32230) at 1:2000 dilution. Images were collected using Odyssey Imager and analyzed using ImageStudio Lite software. A separate group of rats was injected with a single I.P. injection of either Ang II (36 μg/kg) or regular saline vehicle, and perfused at either 1 or 24 h following Ang II injection (*n* = 4 per group), in order to quantify levels of activated microglia (Iba1) and astrocytes (GFAP) in the PVN using established immunohistochemistry (IHC) protocols (Jun et al., 2012; Zubcevic et al., 2014; Santisteban et al., 2015). All Images were taken with Zeiss fluorescence microscope at 10x and 20x. Total number of PVN microglia and astrocytes was established by two blinded researchers using ImageJ as described before (Shi et al., 2010; Santisteban et al., 2015). The PVN was chosed as a focal cardioregulatory region due to its highly vascularized nature, while neuroinflammatory responses in the PVN have previously been linked with chronic Ang II HTN (Dange et al., 2015; de Kloet et al., 2015; Hurr and Young, 2016). In addition, activated microglia was morphologically identified using established techniques (Nimmerjahn et al., 2005; Hains and Waxman, 2006). Activated microglia presented with larger cell bodies and were round-shaped, possessing thicker branches compared to the resting microglia, as previously observed (Nimmerjahn et al., 2005; Hains and Waxman, 2006). Activated microglia was presented as %total microglia in the bilateral PVN at 1 and 24 h following Ang II injections compared to their respective controls.

### Manganese-enhanced magnetic resonance imaging (memri) in the wky rat

Manganese-enhanced magnetic resonance (MEMRI) is a very powerful experimental technique that provides a novel approach for examining *in vivo* neuronal activity in chronic disease models (Yu et al., 2005; Kimura et al., 2007; Perez et al., 2013; Zubcevic et al., 2014; Thinschmidt et al., 2016). The experimental design was performed as previously described (Zubcevic et al., 2014). Manganese (II) chloride tetrahydrate was purchased from Sigma-Aldrich Chemical Co. (St. Louis, MO, USA) and was dissolved in ddH_2_O and sterile-filtered prior to administration. The manganese ion (Mn^2+^) is a calcium ion analog, which is transported across the BBB in a dose-dependent manner and causes signal intensity enhancement within hours, with a peak intensity occurring between 14 and 48 h and a washout by 96 h (Lee et al., 2005; Kimura et al., 2007; Inoue et al., 2011; Jackson et al., 2011). Importantly, entry of Mn^2+^ through voltage-dependent calcium channels at synaptic terminals and neuronal soma leads to accumulation of activity dependent contrast that is visualized in T1 weighted MRI scans. This makes it an ideal approach for examining long-term alterations in neuronal activity associated with neurogenic HTN. Brain images were collected in isoflurane-anesthetized rats on an actively shielded 4.7-Tesla Magnex Scientific MR scanner controlled by Agilent Technologies VnmrJ 3.1 console software. A 38-mm quadrature transmit/receive radiofrequency (RF) coil tuned to 200 MHz was used (Insight NeuroImaging Systems, LLC, Leominster, MA). Prior to imaging, WKY rats (*n* = 6 per group) were injected with either a single I.P. Ang II injection (36 μg/kg) or regular saline vehicle. At the same time, a single I.P. injection of 50 mg/kg of manganese (II) chloride tetrahydrate in 0.5 ml regular saline was administered to all rats. Twenty four hours post injections, anesthesia in all rats was initially induced under 2.0–2.5% isoflurane (0.1 mL/min) delivered in 100% oxygen for 30–60 s, and the levels were maintained with 1.0–1.25% throughout the imaging experiment. Rats were placed in a plastic cylindrical frame with a respiratory monitor pad secured at the level of the diaphragm. The head was fixed in place with ear bars and a bite bar secured to the coil frame to minimize the motion (Santisteban et al., 2013). The head was then placed inside a cylindrical holder with a built-in quadrature transmit/receive volume coil (*AIRMRI, LLC, Holden, MA, USA*). Body temperature was maintained using a warm air recirculating system that received feedback from a fiber optic thermocouple microprobe (SA Instruments, Inc., New York). Respiratory rates were monitored continuously and maintained between 50 and 60 beats per minute by adjusting isoflurane levels. Images were acquired using a T_1_-weighted spin-echo multi-slice sequence with the following parameters: repetition time (TR) = 275.57 ms, echo time (TE) = 16.38 ms, data matrix 256 × 256 (along read × phase directions), size 30 × 30 mm (resolution 117 × 117 μm along the read and phase directions), 12 consecutive slices with 1.2 mm thickness and no gap. The images were averaged 30 times, and the total scan time per rat was 35 min.

### MRI data processing

Scans were aligned with a segmented atlas of the rat brain using an automated affine linear registration tool from FMRIB software library (*flirt* program in FSL, Oxford University). We used a voxel-wise signal normalization procedure as previously published (Perez et al., 2013; Zubcevic et al., 2014; Thinschmidt et al., 2016). Image processing was carried out using itk SNAP (http://www.itksnap.org) and Matlab custom code. A pre-set voxel normalized signal threshold ≥1 was selected based on *a priori* observation of individual datasets and a close inspection of their intensity distribution histograms for rats without and with manganese administration. Normalized voxel intensity values (in Z scores) and the number of voxels equal to or above the threshold value were extracted for the regions of interest (ROIs).

### Quantification of fos levels in the PVN and SFO of wky following acute Ang II injections

Adult male WKY rats (*n* = 6 per group) were injected with either regular saline or Ang II I.P. (36 μg/kg), for quantification of immediate early (Fos) expression levels in order to confirm the neuronal activation in the PVN at 1 and 24 h following Ang II injection, using previously described methods (Krause et al., 2011). Briefly, 1 and 24 h post-injections, animals were anaesthetized and intracardially perfused with ice-cold PBS saline followed by freshly-made ice-cold 4% paraformaldehyde (PFA). Brains were post-fixed in 4% PFA at 4°C overnight, and cryoprotected in 30% sucrose at 4°C prior to sectioning. Free-floating slices were cut in the cryostat at 10 μm and rinsed in cold PBS. For Fos IHC, free-floating brain slices were incubated with blocking solution (2% donkey serum and 0.2% TX-100 in PBS) for 1 h, followed by incubation with a primary anti-c-Fos (Encor Mouse Anti-cFOS, 1:2000) overnight at 4°C in blocking solution. Secondary antibody (donkey anti-mouse Alexa Fluor 647; 1:500) was applied for 2 h at 4°C in blocking solution. Following rinsing with PBS, slices were mounted onto glass slides and cover-slipped using VectaShield. Images were taken with Zeiss fluorescence microscope at 10x and 20x. Total number of Fos-labeled cells in the PVN was separately quantified by two independent researchers. The results were averaged between the two separate counts. Results of Ang II injections at 1 and 24 h were compared to the results of control saline injections performed at the same time and presented as %Control.

### Data and statistical analysis

Data were expressed as mean ± SEM. 2-way ANOVAs or 1-way ANOVAs, and the Bonferroni post-test was used to allow multiple comparisons of cardiovascular variables across time and between different groups. Student *t*-tests were used for comparisons between 2 groups where applicable, with *p* < 0.05 considered significant.

## Results

### Effects of acute Ang II on mean arterial pressure and autonomic nervous system

**Figure 1:**
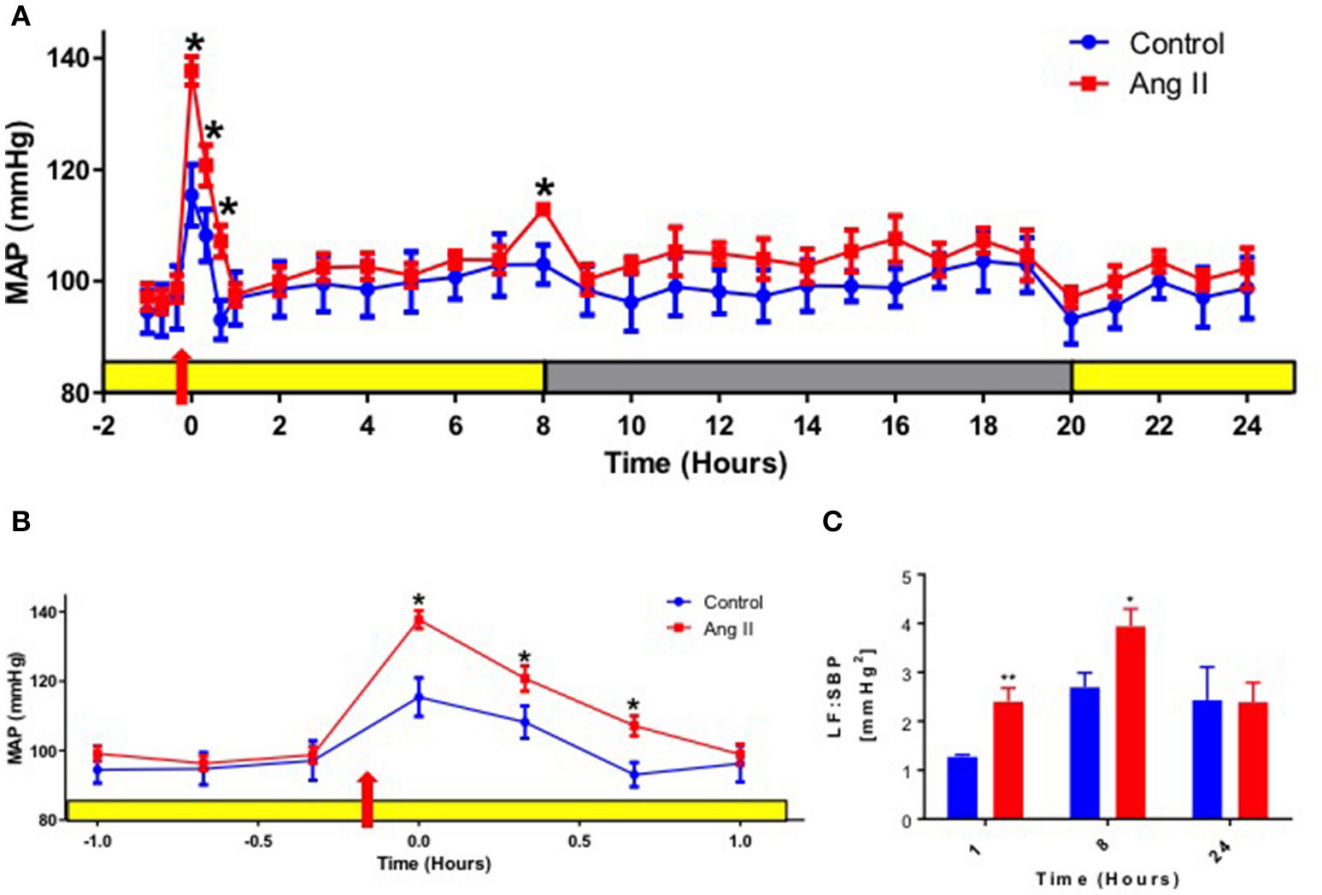
Effects of systemic Ang II injection on blood pressure. **(A)** Mean arterial pressure (MAP) over 24 h in WKY rats injected with Ang II (red) or control saline I.P. (blue). Yellow bar represents daylight period, and gray represents night time period. Red arrow represents time of Ang II injection, 10 min prior to recording at time zero. **(B)** A re-plotted diagram of MAP effects highlighting 1 h prior and post-Ang II (red) or control saline injection (blue). **(C)** Power spectral analysis of systolic blood pressure (SBP) signal at 1,8 and 24 h post Ang II injection (red bars) vs. control saline-injected WKY at the equivalent time points (blue bars). ±SEM. *N* = 6 per group. Unpaired Student *T*test was used in B and C. ^*^p < 0.05 vs. control, ^**^p < 0.01 vs. control.

Single Ang II injection (36 μg/kg, I.P.) in normotensive WKY rats caused a transient increase in mean arterial pressure (MAP) when compared with saline-injected controls, with optimal increase seen within 1 h (117 ± 4 mmHg saline vs. 139 ± 4 mmHg Ang II, *p* < 0.05, Figures 1A,B). The increase in MAP observed in saline-injected rats was attributed to the effect of handling, injection and movement of the rats as they were picked up from their cages, injected and placed back in the cage. The increase in MAP in Ang II injected rats was associated with 1.8-fold elevation of low frequency of systolic blood pressure [(LF:SBP: 1.28 ± 0.03 saline vs. 2.41 ± 0.27 Ang II, *p* < 0.05, Figure 1C). This indicated that MAP increase was associated with elevated vasomotor sympathetic drive even within 1 h. Continuous monitoring of MAP throughout light and dark cycle following a single Ang II injection revealed an interesting pattern. First, MAP returned to baseline and remained at this level throughout the remainder of the light cycle (depicted by yellow bar in Figure 1A). Second, a significant spike of MAP was observed at the commencement of the dark cycle (103 ± 3 mmHg saline vs. 113 ± 0.9 mmHg Ang II, *p* < 0.05) at 8 h post-Ang II administration (depicted by gray bar in Figure 1A). This increase in MAP was also associated with a 27% increase in LF:SBP (Figure 1C), which returned to baseline within 2 h (not shown). This spike in MAP and LF:SBP is a common physiological response at the beginning of the dark cycle in rodents, as their activity increases significantly immediately following the lights-off (Zubcevic et al., 2014). However, the significantly increased MAP and LF:SBP responses in Ang II-injected rats at 8 h post-injection suggest central effects that persisted long after initial Ang II injection, possibly due to priming of the system to subsequent stimuli, such as increased activity. In line with this, ratio of LF:SBP and high frequency component of pulse interval (HF:PI), a measure of vagal balance, remained persistently elevated at 1, 8, and 24 h (Figure S1C). However, no significant changes in either HF:PI or spontaneous baroreflex gain (SBRG:PI) were observed (Figure S1). Lastly, both MAP and LF:SBP returned to baseline levels for the remainder of the dark cycle and up to 24 h of experimental measurements.

### Persistent systemic and neuro-immune activation by acute Ang II

**Figure 2:**
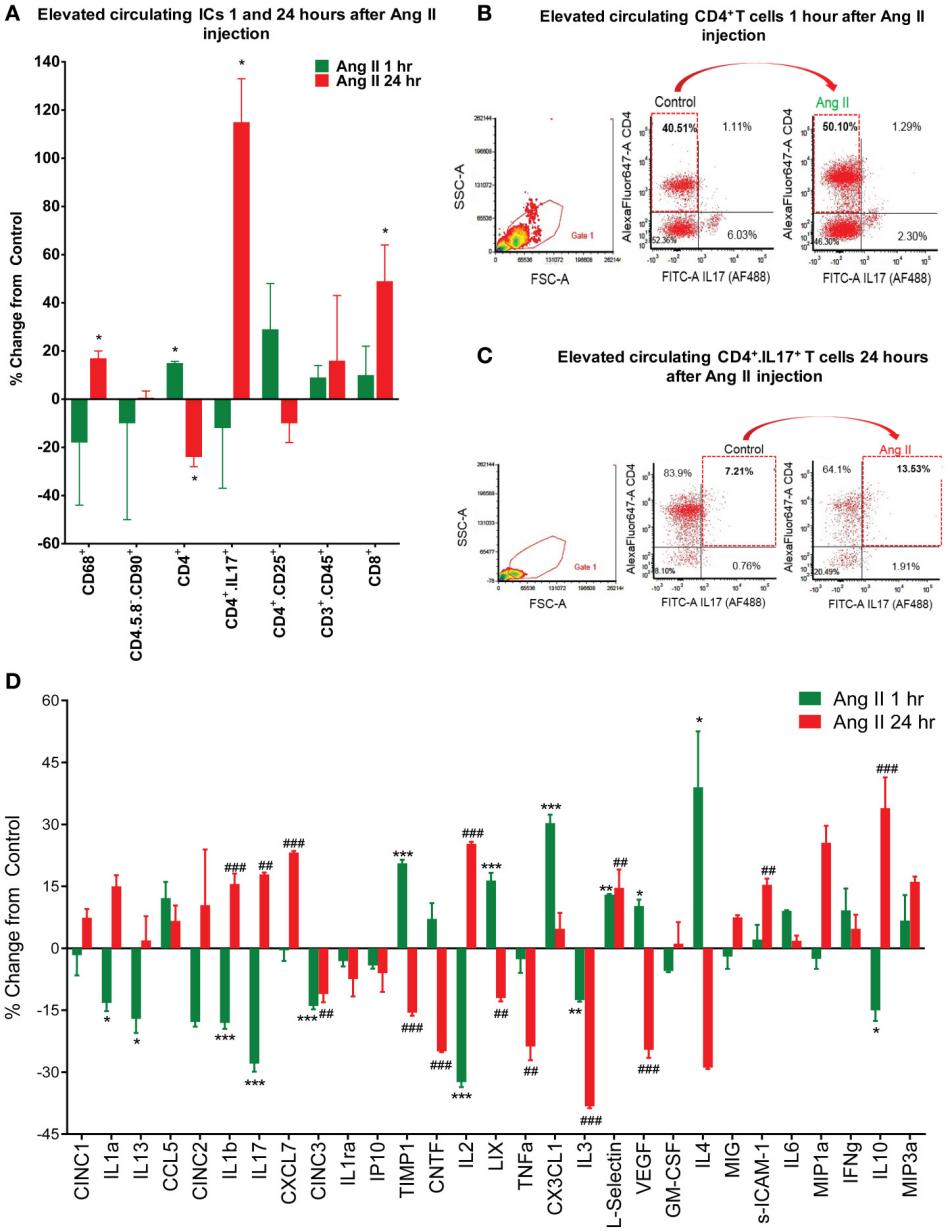
Effects of systemic Ang II injection on peripheral and central immune response. **(A–C)** Quantification of circulating inflammatory cells show an increase in CD4^+^ T cells even at 1 h post-Ang II injection **(A,B)** and persistence of CD4^+^/IL17^+^ T cells, amongst others, at 24 h post-Ang II injection **(A,C)**. In **(D)**, changes in PVN cytokine levels following a single Ang II injection I.P., as measured by ELISA at 1 h (green bars) and 24 h (red bars) following the injection. ±SEM. *N* = 46 per group, ^*^P < 0.05, ^**^^##^*P* < 0.01, ^***^^###^*P* < 0.001, vs. respective control. ICs, inflammatory cells.

**Figure 3:**
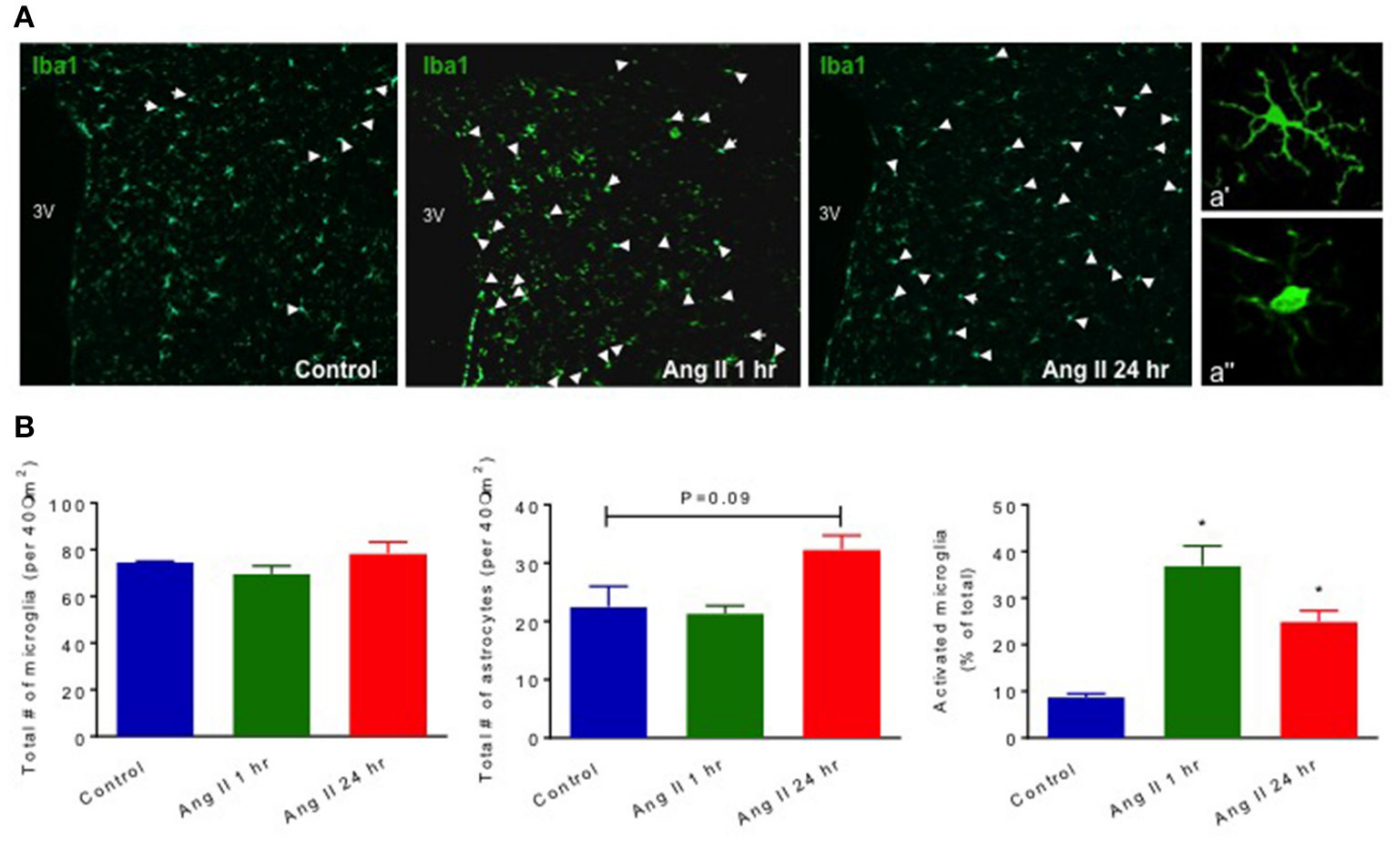
Effect of systemic Ang II injection on microglial activation and astrocytes in the PVN. Quantification of Iba1^+^microglial cells in the PVN of WKY shows an increase in activated microglia at 1 and 24 h following Ang II injection (in **B**, far right). Quantification of total GFAP^+^ astrocytes following Ang II injections showed a trend in increase at 24 h post-Ang II-injection (in **B**, middle graph). Representative images of PVN microglial cells at three different time points are shown in **(A)**. White arrow points to activated microglial cells in Control, and 1 and 24 h following Ang II injections. Higher magnification (40x) images of representative resting and activated microglia are shown in a' and a” respectively. ±SEM. *N* = 4 per group, ^*^p < 0.05 vs. control.

Our previous studies have established that chronic Ang II infusion causes HTN which involves microglial activation and increased inflammatory cytokines in autonomic brain regions, primarily in the PVN, as well as increased systemic inflammation and dysfunctional ANS (Shi et al., 2010; Jun et al., 2012). In this study, we tested if a single dose of Ang II, that does not cause HTN, is sufficient to induce neuro- and peripheral inflammation. First, we compared circulating ICs and PVN cytokines between saline- and Ang II-treated rats, to confirm the inflammatory status. Circulating levels of CD4^+^ T cells were increased 1 h following Ang II treatment, while CD4^+^/CD25^+^, CD3^+^/CD45^+^ and CD8^+^ T cells showed an upward trend (*p* < 0.05, Figure 2A, green bars). At 24 h, we observed a ~110% increase in CD4^+^/IL17^+^ T cells and a ~50% increase in CD8^+^ T cells (*p* < 0.05, Figure 2A, red bars), suggesting delayed activation of peripheral IS. Figures 2B,C show representative examples of raw FACS readings for CD4^+^/IL17^+^ ICs at 1 and 24 h respectively. Elevated levels of PVN inflammatory mediators were observed both at 1 h and 24 h, although the composition varied. For example, significant increases in CX3CL1, L-selectin, and IL4 (*p* < 0.05, green bars) were observed at 1 h; while increases in CXCL7, L-selectin, s-ICAM-1 and IL10 (*p* < 0.05, red bars) were observed at 24 h (Figures 2D, S2). In addition, a subset of inflammatory mediators showed a biphasic response, with significant increase at one time point and a decrease at other (e.g., TIMP 1, IL2, LIX, IL1β, IL17, and VEGF, *P* < 0.05). We also observed increased proportion of activated microglia at 1 h (by 400%, *P* < 0.05), which persisted at 24 h (by 277%, *P* < 0.05) compared to control (Figure 3). Taken together, these observations indicate that even a single Ang II administration produced prolonged systemic and neuroinflammatory responses that seem to be independent of high BP.

### Prolonged increase in neuronal activity in autonomic brain nuclei by acute Ang II

**Figure 4:**
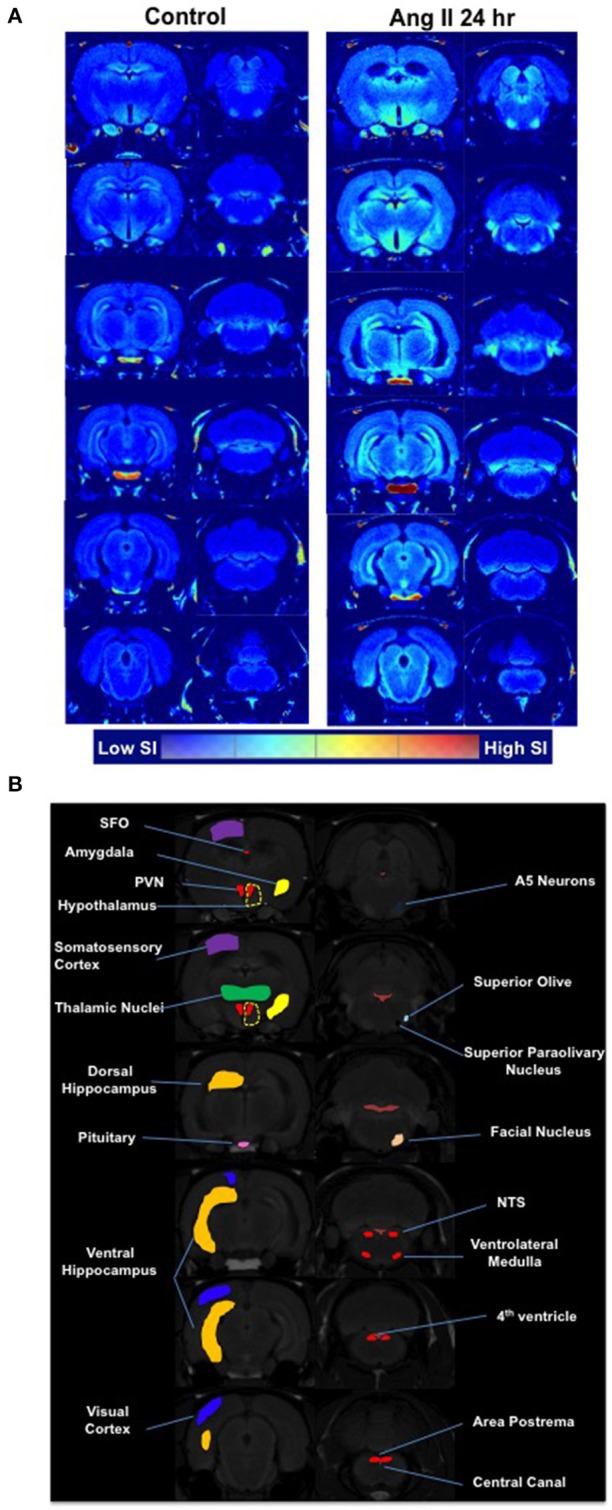
Effects of systemic Ang II injection on the brain. **(A)** A single I.P. injection of Ang II caused a marked increase in the manganese-enhanced signal, corresponding to neuronal activation, in several nuclei of the forebrain and hindbrain as measured by MEMRI 24 h following Ang II injection (right panel) and compared to saline injection (left panel) (averaged from *n* = 6 per group). The brain segmentation slices are arranged consequentially from the brainstem regions (bottom left segment) through to the hypothalamic PVN region (bottom right segment). **(B)** Map of brain regions as per Paxinos-Watson.

**Figure 5:**
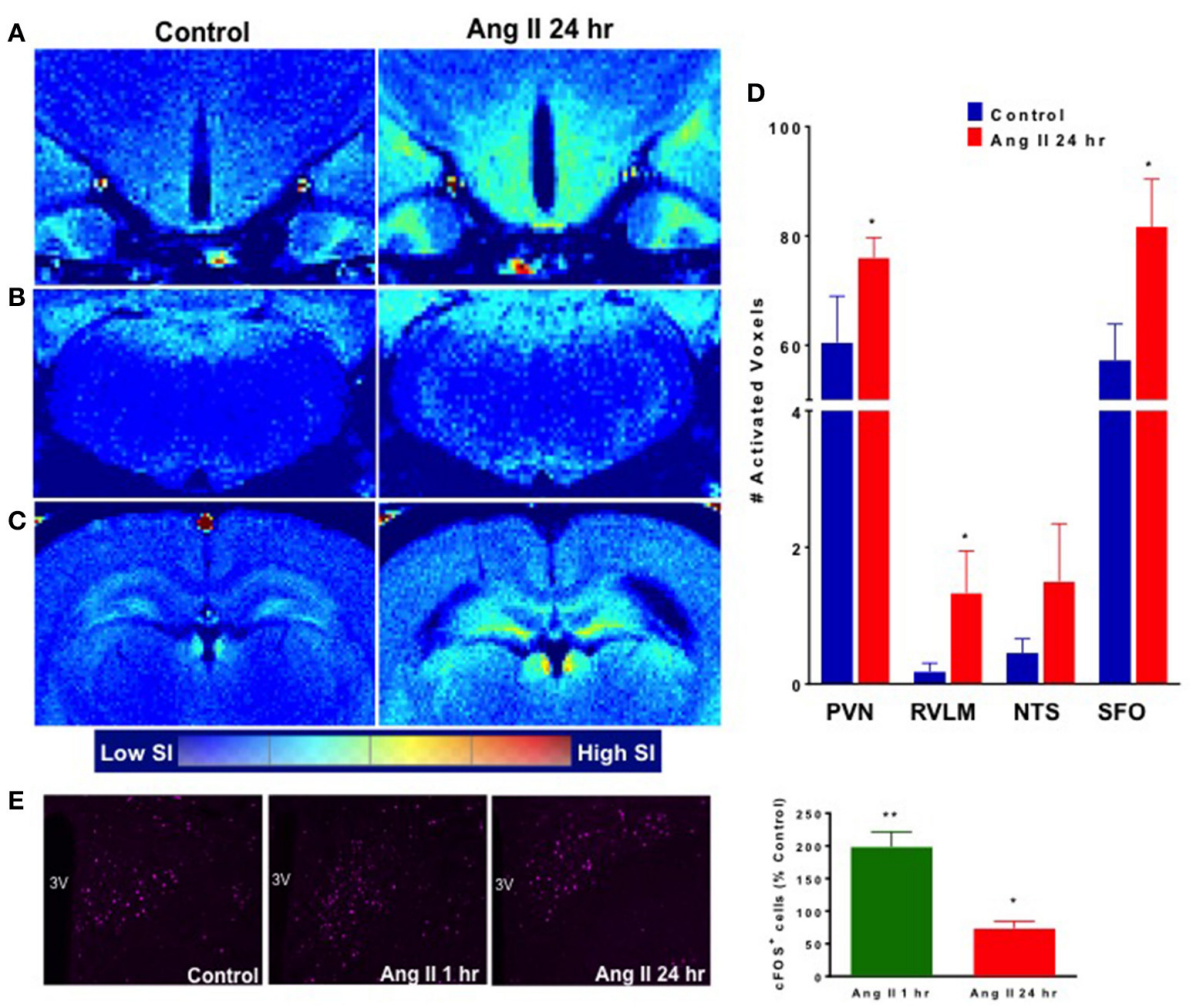
Effect of systemic Ang II injection on cardioregulatory brain regions. Manganese-labeled hypothalamic **(A)**, brainstem **(B)** and circumventricular **(C)** activity following control injection (left) and 24 h following Ang II injection (right). **(D)** Quantification of activated voxels in the PVN, RVLM, NTS and SFO, 24 h following Ang II injection (red bars). **(E)** Quantification of Fos expression in the PVN (purple) at 1 and 24 h following Ang II injection. ±SEM. *N* = 4–6 per group, Unpaired Student Ttest was used in D and E. ^**^p < 0.01, ^*^p < 0.05 vs. control.

Manganese-enhanced magnetic resonance (MEMRI) was used to determine activity of neurons in autonomic brain nuclei 24 h following Ang II administration. The technique utilizes manganese (Mn^2+^) sequestration in actively-firing neurons, but it limits us to measure the activity only at longer time periods, as peak of Mn^2+^ sequestration occurs within hours following its application (Lee et al., 2005; Inoue et al., 2011). We observed a marked increase in the manganese-enhanced signal, corresponding to neuronal activation, in several nuclei of the forebrain and hindbrain that persisted at 24 h post-Ang II-injection. Representative images are shown in Figure 4. More specifically, we observed significant increases in neuronal activation in the PVN (~20%), rostral ventrolateral medulla (RVLM, ~900%) and subfornical organ (SFO, ~25%) 24 h post-manganese injection (Figures 5A–D). Other cardio-relevant regions that showed a significant increase in neuronal activity at 24 h following the Ang II injections were as follows: central amygdaloid nucleus (cAN), lateral hypothalamus (LHN), median raphe nucleus (mRN), parabrachial nucleus (PN), supraoptic nucleus (SON), and sub coeruleus nucleus (SCN), while many other showed a similar trend (Figure S3). In addition, significant manganese-enhanced signal was observed in the pituitary, and more appropriately, the Sella turcica, which was attributed to its highly vascularized nature, which leads to higher accumulation of manganese (Figure 4). Thus, we use this region to confirm successful injections of manganese in rats. Lastly, Fos immunoreactivity was used to confirm PVN neuronal activity (Figure 5E). As expected, the total number of Fos-positive cells increased by ~2-fold at the hour following Ang II administration (green bar, Figure 5E). Moreover, the number of Fos-positive cells remained significantly higher (by 73%, *p* < 0.05, red bar, Figure 5E) at 24 h.

## Discussion

The major findings of the present study are as follows: (1) Even a single Ang II challenge is able to produce significant effects on the IS and CNS that persist after the BP is normalized. (2) These effects extend to activation of central IS, beginning as early as 1 h and continuing over 24 h following the Ang II challenge. Thus, to our knowledge this is the first study to suggest a role for a single pro-hypertensive Ang II challenge in initiation of inflammatory and neural changes. These findings contribute to understanding of the early communication that exists between RAS, ANS, and IS, three major players in the development and maintenance of HTN.

Johnson et al have proposed a “sensitization” concept in neural dysregulation in HTN. They have established that subpressor doses of chronic Ang II infusion, administered continuously over 1 week, enhance BP responses to subsequent pro-hypertensive challenges with Ang II or aldosterone (Xue et al., 2012). This sensitization of the hypertensive responses by Ang II is mediated via the CNS, suggesting a form of neuroplasticity in the context of neurogenic hypertension (Clayton et al., 2014). This form of response sensitization is part of a normal neurobiological response previously associated with many mechanisms including the baro- and chemoreflex, intermittent hypoxia, stress, exercise, salt appetite etc. (Mifflin, 1997; Na et al., 2007; Herman et al., 2008; Kline, 2008; Cunningham et al., 2012), as healthy systems attempt to adapt to various environmental and humoral factors in order to preserve homeostasis. However, this capacity for reprogramming can cause CNS maladaptation toward pathological in the presence of continuous challenges and stressors. Our observations are somewhat consistant with this concept. However, they are unique in a way that we, for the first time, show that even an acute challenge by Ang II, at a dose that is reportedly subpressor when applied chronically (Yasujima et al., 1989) may be able to induce an adaptive BP response that manifests several hours post-challenge and sensitize the neural pathways. This response appears to be centrally mediated, demonstrated by significant increase in LF:SBP, a derived measure indicative of changes in the sympathetic vasomotor drive (Waki et al., 2006), immediately post-Ang II injection, and then again at 8 h post-Ang II challenge. This time point at 8 h following our initial Ang II challenge marks the very beginning of the dark and most active cycle for rodents (Hurwitz et al., 1985). Thus, the significantly higher MAP and LF:SBP response in Ang II-injected rats at 8 h following the initial challenge may be associated with sensitization of BP and LF:SBP responses to secondary pro-hypertensive challenge at this point, in this case an elevation in sympathetic drive and/or release of additional pro-hypertensive stressors that are known to accompany the beginning of the dark phase in rodents (Hurwitz et al., 1985). As we observed no change in the parasympathetic HF:PI variable at any measured time point, the activation of central neural pathways as observed by MRI may not reflect activation of blood pressure-correcting neural mechanisms, and may indeed be specific to activation of the Ang II-sensitized pre-sympathetic neurons. Circulating Ang II is known to activate AT1 receptors at the circumventricular organs to stimulate downstream neural pathways including those to SFO and PVN (Cancelliere et al., 2015; Coble et al., 2015). However, although we did not measure the circulating levels of Ang II in our study, it is unlikely that Ang II was present in circulation at eight or 24 h following injection, considering its short half-life (Al-Merani et al., 1978; Chapman et al., 1980). Keeping in mind that studies by Johnson et al describe BP sensitization responses following chronic Ang II, we are aware that mechanisms at play here may be different. However, considering the increased neural activation in several cardioregulatory brain regions, as evidenced by MRI and Fos immunohistochemistry performed 24 h post-Ang II challenge, we propose that even a single Ang II challenge may produce priming effects on the ANS.

This study points out early pro-hypertensive neuronal imprinting and raises an important question: does Ang II have a direct ANS/IS effect, or is the increase in MAP one that drives the observed responses at 24 h? The latter is less likely, considering that we have previously shown no changes in the IS response even after 6 weeks of phenylephrine-induced HTN, while comparable chronic Ang II infusion induced HTN while also activating both peripheral and central IS (Jun et al., 2012). Further studies are needed to pinpoint the exact mechanisms, but it is tempting to suggest that the delayed autonomic responses observed in the present study may be reinforced by the presence of peripheral and central inflammation. Although we did not test the neuronal effects of increase in PVN cytokines, others have previously shown that inflammatory cytokines may be involved in central regulation of BP (Takagishi et al., 2010; Gouraud et al., 2011). Systemically, we observed that the immediate Ang II response was associated with an increase in circulating inflammatory CD4^+^ T cells, a hallmark of HTN (Guzik et al., 2007; Jun et al., 2012). Centrally, we observed an immediate activation of microglia and elevation in several inflammatory mediators in the PVN: CX3CL1, LIX, TIMP 1 and IL4, all markers of microglial activation (Nuttall et al., 2007; Franco and Fernandez-Suarez, 2015; Jha et al., 2016; Li et al., 2016). Delayed responses are particularly reflected in strengthening of the Th17 cell phenotype, as we observe a dramatic increase in circulating CD4^+^/IL17^+^ cells, accompanied by an increase in IL17 cytokine in the PVN 24 h following the Ang II challenge. Recently, accumulation of IL17-releasing T cells in the kidney has been associated with renal and vascular dysfunction in animal models of HTN (McMaster et al., 2015; Itani et al., 2016b; Norlander et al., 2016), while mice deficient in IL17 are protected against Ang II HTN (Madhur et al., 2010). Furthermore, IL17 is able to induce expression of a host of HTN-associated inflammatory cytokines (Madhur et al., 2010), and is linked to breakdown of BBB in animal and human disease (Kebir et al., 2007; Huppert et al., 2010). Although we did not measure the integrity of BBB in the present study, others have suggested that chronic applications of Ang II are needed to compromise its function (Biancardi et al., 2014; Biancardi and Stern, 2016; Faraco et al., 2016). Most interestingly, a recent study by Harrison and colleagues showed that a repeated Ang II stimuli caused expansion of specific IL17-expressing memory T cells, which are proposed to sensitize the host to the development of HTN in response to subsequent mild pro-hypertensive stimuli, thus creating a form of immunological memory (Itani et al., 2016b). Here, we show that even a single Ang II challenge can elevate the IL17-expressing T cells in circulation, and increase the levels of IL17 cytokine in the PVN. Thus, we propose that immediate effect of Ang II in our study may be central, leading to direct neuronal and microglial activation and consequent increase in MAP and LF:SBP at 1 hour. The delayed Ang II-dependent effects may be fortified by activation of the sympathetic drive, and support a role for activated peripheral inflammatory cells, and especially those expressing IL17, that may continue central neural sensitization for hours following the initial Ang II challenge. Further studies are required to confirm or refute this hypothesis.

Although not all individuals will develop HTN, according to the CDC, 1 in 3 individuals in the US are hypertensive, while additional 1 in 3 individuals are borderline hypertensive (Merai et al., 2016). Moreover, AHA report states that 66–80% individuals that are 75 and older suffer from HTN (Mozaffarian et al., 2015). Thus, the prevalence of this condition increases with age. In terms of the results of the current study as well as similar studies by others (Johnson et al., 2015), accumulation of many pro-HTN insults with time could tip the balance toward the disease. Naturally, the timeline and severity of development of the condition may be dependent on many factors, environmental and/or genetic.

## Summary

In summary, even a single systemic Ang II injection can activate both the peripheral and central IS, and affect the autonomic function in our model. The questions remain whether these responses are initiated centrally or peripherally, and if they are primarily ANS- or IS-mediated. Considering the short timeline, the present techniques limit our current conclusions, and further studies are needed to better dissect the mechanisms of early RAS-ANS-IS interaction in the context of development of neuronal and immune memory in HTN.

## Author contributions

JZ designed and performed the experiments and contributed to writing of the manuscript. MS performed telemetry experiments and analyzed the data. PP performed MRI and analyzed the data. RA performed staining and analyzed the data. HH performed staining and analyzed the data. WM performed FACS and analyzed the data. LC analyzed MRI data. RS performed confocal imaging. AdK analyzed staining experiments. EK analyzed staining experiments and edited the manuscript. MF oversaw MRI experiments, analyzed data, made figures and edited the manuscript. MR designed experiments, edited and contributed to writing of the manuscript.

### Conflict of interest statement

The authors declare that the research was conducted in the absence of any commercial or financial relationships that could be construed as a potential conflict of interest.

## Supplementary material

Figure S1 Power spectral analysis of SBP signal at different time points following Ang II (red bars) and control saline injections (blue bars) in the WKY. **(A)** Very low frequency (VLF) of SBP. **(B)** High frequency (HF) of pulse interval (PI). **(C)** Low frequency (LF) to high frequency (HF) ratio. **(D)** Spontaneous baroreflex gain (sBRG) of pulse interval (PI). *N* = 6 per group, ^*^*P* < 0.05.

Figure S2 ELISA plate showing the individual results for cytokines measured in the PVN at 1 h **(A)** and 24 h **(B)** post saline and Ang II injections.

Figure S3 Changes in the manganese-labeled activation of several cardioregulatory brain regions following a single systemic injection of Ang II (red bars). *N* = 6, ^*^*P* < 0.05. AHA, anterior hypothalamic area; cAN, central amygdaloid nucleus; dMN, dorsal medial nucleus; dRN, dorsal raphe; lAN, lateral amygdaloid nucleus; LC, locus ceruleus; LHN, lateral hypothalamus; mAN, medial amygdaloid nucleus; mRN, median raphe nucleus; PN, parabrachial nucleus; PHA, posterior hypothalamic area; RL, raphe linear; SNC, substantia nigra compacta; SON, supraoptic nucleus; SCN, sub coeruleus nucleus; VS, ventral subiculum.
