# Supplementary figures and images for: A Single Angiotensin II Hypertensive Stimulus Is Associated with Prolonged Neuronal and Immune System Activation in Wistar-Kyoto Rats
Source: Front Physiol. 2017 Aug 31;8:592. doi: 10.3389/fphys.2017.00592 (PMC5583219; doi:10.3389/fphys.2017.00592)

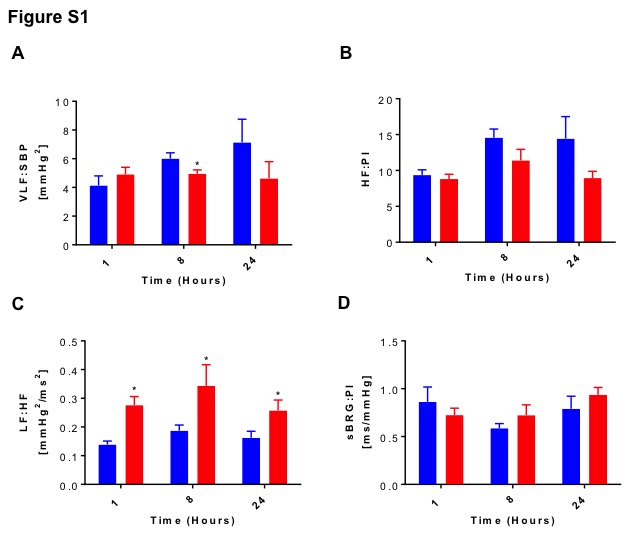

Supplement: Figure S1 — Power spectral analysis of SBP signal at different time points following Ang II (red bars) and control saline injections (blue bars) in the WKY. (A) Very low frequency (VLF) of SBP. (B) High frequency (HF) of pulse interval (PI). (C) Low frequency (LF) to high frequency (HF) ratio. (D) Spontaneous baroreflex gain (sBRG) of pulse interval (PI). N = 6 per group, *P < 0.05. [file Image1.jpeg]

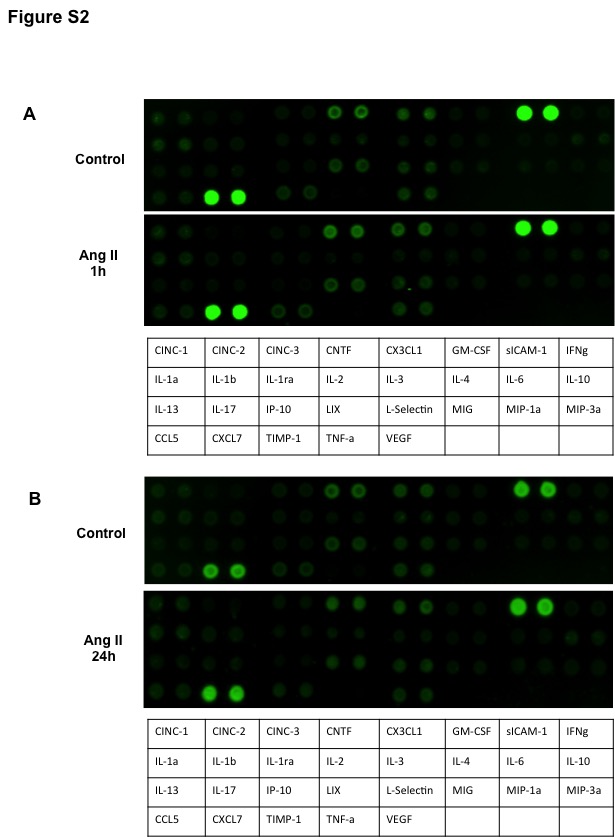

Supplement: Figure S2 — ELISA plate showing the individual results for cytokines measured in the PVN at 1 h (A) and 24 h (B) post saline and Ang II injections. [file Image2.jpeg]

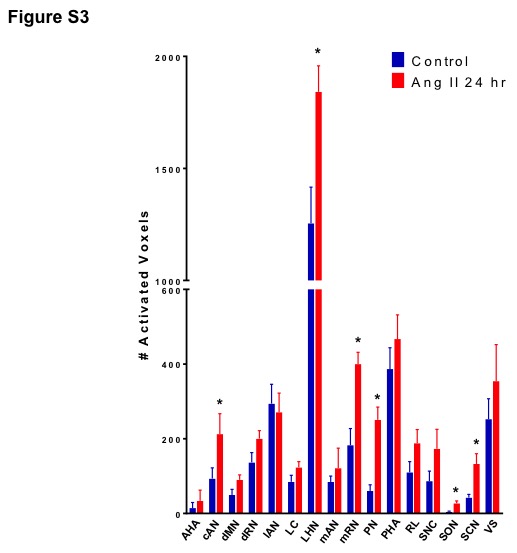

Supplement: Figure S3 — Changes in the manganese-labeled activation of several cardioregulatory brain regions following a single systemic injection of Ang II (red bars). N = 6, *P < 0.05. AHA, anterior hypothalamic area; cAN, central amygdaloid nucleus; dMN, dorsal medial nucleus; dRN, dorsal raphe; lAN, lateral amygdaloid nucleus; LC, locus ceruleus; LHN, lateral hypothalamus; mAN, medial amygdaloid nucleus; mRN, median raphe nucleus; PN, parabrachial nucleus; PHA, posterior hypothalamic area; RL, raphe linear; SNC, substantia nigra compacta; SON, supraoptic nucleus; SCN, sub coeruleus nucleus; VS, ventral subiculum. [file Image3.jpeg]
